# Rapidoxy® 100: A Solvent-Free Pre-treatment for Production of Canolol

**DOI:** 10.3389/fnut.2021.687851

**Published:** 2021-07-02

**Authors:** Ruchira Nandasiri, Afra Imran, Usha Thiyam-Holländer, N. A. Michael Eskin

**Affiliations:** ^1^Department of Food and Human Nutritional Sciences, University of Manitoba, Winnipeg, MB, Canada; ^2^Richardson Centre for Functional Foods and Nutraceuticals, Winnipeg, MB, Canada

**Keywords:** RapidOxy® 100, canola meal, canolol, high temperature, time, inert-atmosphere, mustard (*Brassica juncea*), canola (*Brassica napus* L.)

## Abstract

RapidOxy® 100 is an automated instrument originally designed for measuring the oxidative stability of both solid and liquid samples. The compact and portable design of RapidOxy® 100, and its built-in pressurized heating chamber, provides a suitable environment for studying processing conditions. The feasibility of using oxygen or an inert atmosphere provides the ideal environment to study the effect of dry heat pre-treatment on canola antioxidants. The current study used RapidOxy® 100 to examine the impact of pressurized dry heat pre-treatment, under nitrogen, on the ultrasonic extraction of phenolic compounds. The effect of different pre-treatment temperature-time combinations of 120, 140, 160, and 180°C for 2, 5, 10, 15, and 20 min on the subsequent extraction of canola phenolic compounds was examined. The major sinapates identified by HPLC were sinapine, sinapic acid, and canolol. The optimum RapidOxy® condition for the maximum recovery of canolol was 160°C for 10 min. RapidOxy® 100 proved to be a novel and versatile instrument for enhancing the extraction of phenolic compounds.

## Introduction

*Brassica* family comprise a wide range of horticultural and agricultural crops that are extensively used worldwide. Of these canola (*Brassica napus* L.) and mustard (*Brassica juncea* L.) are among the leading oil crops worldwide. The high demand of oils from these crops produces large amounts of meal and cake by-products. These by-products are limited to the animal industry as feed ingredients and as such are underutilized (1, 2). Although, the meal contains many functional ingredients including amino acids, and phenolic compounds it has gained less attention over the last few decades due to many anti-nutritive factors including glucosinolates (2). However, its content of minor components including the phenolic compounds are highly valued for their health promoting properties and provide an economic incentive for the greater utilization of canola meal. The meal contains both free and esterified forms of phenolic compounds. Sinapine, an ester of sinapic acid and choline, accounts for over 80% of the phenolic composition (3, 4). Apart from sinapine, sinapic acid, other flavanol compounds including kaempferol, and kaempferol derivatives (mono-, di-, tri- glucosides) are also present in the meal (4–7).

The concentration of phenolic compounds is affected by processing conditions, including high pressure and temperature (6, 7). The application of higher temperatures and pressures often generates novel phenolic compounds with high antioxidant activity such as canolol, its dimers, oligomers, and other breakdown products (8). Moreover, the extractability of major sinapates can be improved with the application of high temperature (~200°C), and pressure (~2,000 psi) (9). The application of high temperature, and pressure reduces the surface tension and viscosity of the extracting solvents and enhances the solubility and mass transfer of targeted phenolics, a key advantage of the pressurized temperature processing (9).

The application of high pressure and temperature, however, requires special equipment and is generally associated with higher operational costs. Hence, there is a need for economical and simple extraction techniques with a higher rate of precision for use by the oilseed processing industry. The extractability of phenolic compounds primarily depends on the polarity of the particular extracting solvent used (10). Application of pressurized heat was considered a feasible option to extract both sinapine and sinapic acid due to its moderately high H-bonding donor and accepting capability (6). Previous studies on extractability of phenolic compounds demonstrated that a solvent concentration of 70% (v/v) was the optimum extractant concentration for obtaining phenolic compounds with higher antioxidant activity compared to the corresponding lower extractant concentrations of 60, 40, and 30% (v/v) (6, 11). Our recent study found that the total phenolic content (TPC) increased with the extraction temperature reaching a maximum at 180°C (*p* > 0.05) with the 70% (v/v) extractant (20.72 mg SAE/g DM) (9).

A newly developed automated instrument RapidOxy® 100 is specifically designed to determine the oxidative stability of various products including foods, cosmetics, flavors and pharmaceutical products in both solid and liquid form. The compact and portable design of RapidOxy® 100, and its built-in pressurized heating chamber, provides the perfect environment for processing conditions. Automated targeted heating (0–180°C) and pressurization up to 500 psi provides the ideal setting to pre-treat canola meal prior to the extraction phenolic compounds. Hence, the substitution of the air supply from oxygen with nitrogen provides an additional advantage by preventing oxidation of the phenolics prior to extraction.

The RapidOxy® 100 instrument provides a unique opportunity to examine the effect of pressure (100 psi) and temperature on the structure-based activity of phenolic compounds. Replacing the air supply with nitrogen (N_2_) would provide an inert pressurized environment, as our previous research showed that such an environment with wet heat was favorable for extracting canolol (9). Our most recent finding also confirmed that 70% methanol (v/v) was optimal for extracting hydroxycinamic acid derivatives (9). The current study examined the structure-based activity of phenolic compounds using RapidOxy® 100 as affected by pressure (100 psi) and temperature. The inert pressurized (100 psi) environment was obtained by replacing the air supply of oxygen with nitrogen. Our previous research demonstrated that an inert pressurized environment with wet heat was favorable for extracting primarily canolol (9). Furthermore, our most recent findings confirmed that an extractant concentration of 70% (v/v) was optimal for extracting hydroxy cinnamic acid derivatives (9).

The current study determined the optimum pre-treatment conditions for the dry-heat extraction of the major canola sinapates, sinapine, sinapic acid, and canolol using a pressurized (100 psi) temperature extraction with the aid of RapidOxy® 100. Four different temperatures-time regimens (120, 140, 160, 180°C and 2, 5, 10, 15, 20 min) were selected under the same high pressure of (100 psi) for the extraction of these major sinapates from both canola (*Brassica napus* L.) and mustard (*Brassica juncea* L.). All solvent extractions were conducted with a 70% (v/v) methanol solution.

## Materials

Mechanically crushed (double expeller pressed) canola meal was provided by Viterra group (St. Agathe, MB, Canada). Both Oriental mustard powder (OMP) and Oriental Mustard Cake (OMC) were provided by G.S Dunn Limited (Hamilton, ON, Canada). Sinapic acid (purity > 98%) was purchased from Fisher scientific Canada Ltd (Ottawa, ON, Canada). Sinapine (purity > 97%) and canolol (purity > 97%) were purchased from ChemFaces® Biochemical Co., Ltd. (Wuhan, Hubei, China). All the HPLC grade solvents other chemicals were purchased from Fisher scientific Canada Ltd (Ottawa, ON, Canada).

## Methods

### Sample Preparation

Canola meal was first ground into powder using a coffee grinder and stored at −20°C until further analysis. Ground canola meal samples were defatted using the Soxtec 2050 as described by Khattab et al. (1) (Foss-Tecator, Foss North America, Eden Prairie, MN, United States).

### RapidOxy® 100 Pre-treatment

RapidOxy® 100 (Anton Paar Canada Inc., Montreal, QC, Canada) was used to determine the optimum temperature-time, pre-treatment condition to extract antioxidative phenolic compounds. A constant pressure (100 psi) was maintained in a user-defined program throughout the pre-treatment duration. For each pre-treatment regime, 1.0 g of defatted canola meal sample was used. Different pre-treatment temperatures (120, 140, 160, and 180°C) were applied to examine the impact of temperature on extractability of phenolic compounds. The test durations (2, 5, 10, 15, and 20 min) were defined in the program for each sample at each of the different temperatures. The stability of each sample was monitored through a specific constant induction time period of 5 min. The inert environment was maintained throughout the experiment with the continuous supply of N_2_ gas to the measuring chamber. Pre-heat-treated canola meal samples were then extracted using the ultrasound for each temperature-time pre-treatment.

### Ultrasonic Extraction

The ultrasonic extraction of phenolic compounds was conducted according to the method described in Liang et al. (12). Briefly, each pre-heat-treated meal sample (1.0 g) was extracted three times with 9.0 mL of methanol (70%, v/v) using a SONOPLUS ultrasonic homogenizer HD 2200 system (BANDELIN electronic GmbH & Co. KG, Heinrichstraße, Berlin, Germany). The ultrasound extraction was carried out at the power of 40% with a frequency of 20 kHz ± 500 Hz for 1 min at room temperature (25°C). After ultrasonic extraction, extracts were centrifuged at 5,000 rpm for 15 min at 4°C (Sorvall Biofuge Primo R Centrifuge; Thermo Scientific, Asheville, NC, USA). The extracts obtained from the three extraction steps were combined and made up a total volume of 30.0 mL.

### HPLC Analysis

The changes in major sinapates obtained from the pre-heat-treated and extracted canola meal samples were evaluated by High Performance Liquid Chromatography (HPLC) described by Nandasiri et al. (9). Phenolic compounds were analyzed by reversed-phase High Performance Liquid Chromatography-Diode Array Detection (HPLC-DAD) (Ultimate 3000, Dionex, Sunnyvale, Torrance, CA, United States). The separation was carried out on a Kinetex® Biphenyl C_18_ 100 Å RP column (2.6 mm, 150 × 4.6 mm, Phenomenex, Torrance, CA, United States), with flow rate of 0.4 mL/min and 10 μL injection volume. The column oven was maintained at 30°C. Extract of 70% aqueous methanol was used in the analysis. Using the authentic standards (sinapine, sinapic acid, and canolol) phenolic compounds were identified. The separation was conducted using gradient elution with water [0.1% (v/v) formic acid] as solvent A, and methanol [0.1% (v/v) formic acid] as solvent B. The chromatograms were acquired at 270 nm (canolol) and 330 nm (sinapine and sinapic acid) using Chromeleon software Version 7.2 SR4 (Dionex Canada Ltd., Oakville, ON, Canada).

### LC-MS Analysis

Structural elucidation of kaempferol-3-*O*-(2*α*-*O*-sinapoyl-*β*-sophoroside), kaempferol-3-*O*-sophoroside, syringic acid, sinapic acid dimer, and methyl sinapate were tentatively identified by liquid chromatography with mass spectrometry and tandem mass spectrometry (LC-MS) using the method described by Nandasiri et al. (13). Cured extracts were dried (N_2_) and analyzed by ESI-MS-MS/MS. Positive ion mode (ESI^+^) was used, and spectra recorded on a Bruker Compact high resolution quadrupole time of flight mass spectrometer (Q-TOF-MS) (Bruker Daltonics, Billerica, Massachusetts, USA). MS mode was applied during the formula generation and the mass range was from 50 to 2,500 m/z was used.

Mass spectrometer was operated at following conditions. The elute pump was operated at a maximum pressure of 10,150 psi, with a capillary voltage of 3,500 V at a dry gas flow rate of 4.0 L/min. Drying temperature was set to 200°C. MS/MS tuning conditions was carried out with ion energy of 5.0 eV and collision energy of 10.0 eV. The fragmentation patterns obtained were compared with the literature values (9, 14–16).

### Statistical Analysis

All the experiments were conducted in triplicate. Results were presented as mean ± standard deviation of triplicate analysis. Data points were checked for their normality and required transformations were carried out to obtain normalized data (17). For the current experiment, logarithmic and square-root transformations were conducted accordingly to obtain normalized data (17). To establish the optimum extraction conditions response surface methodology (RSM) was used. RSM is a well-established statistical technique for obtaining optimal responses with a minimal number of variables (18, 19). RSM also provides detailed information on interaction effects between individual parameters to discover a stationary point (18, 19). Hence, the mathematical models proposed by RSM requires analysis of variance (ANOVA) to determine its adequacy and significance. Statistical analysis was performed using the package “RSM” (20) within the R statistical software version 3.6.0 [R (21)].

## Results and Discussion

### Establishing the Optimized Extraction Conditions for Major Sinapates Using RapidOxy® 100 as a Pre-treatment Method

**Figure 1:**
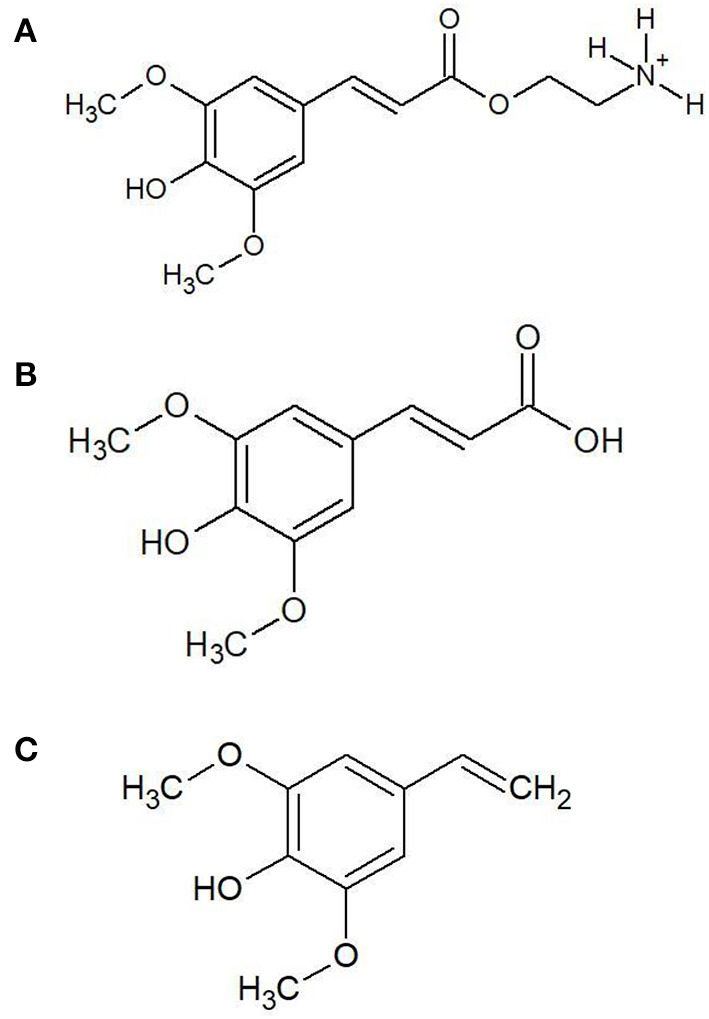
The chemical structures of sinapine **(A)**, sinapic acid **(B)**, and canolol **(C)**.

**Figure 2:**
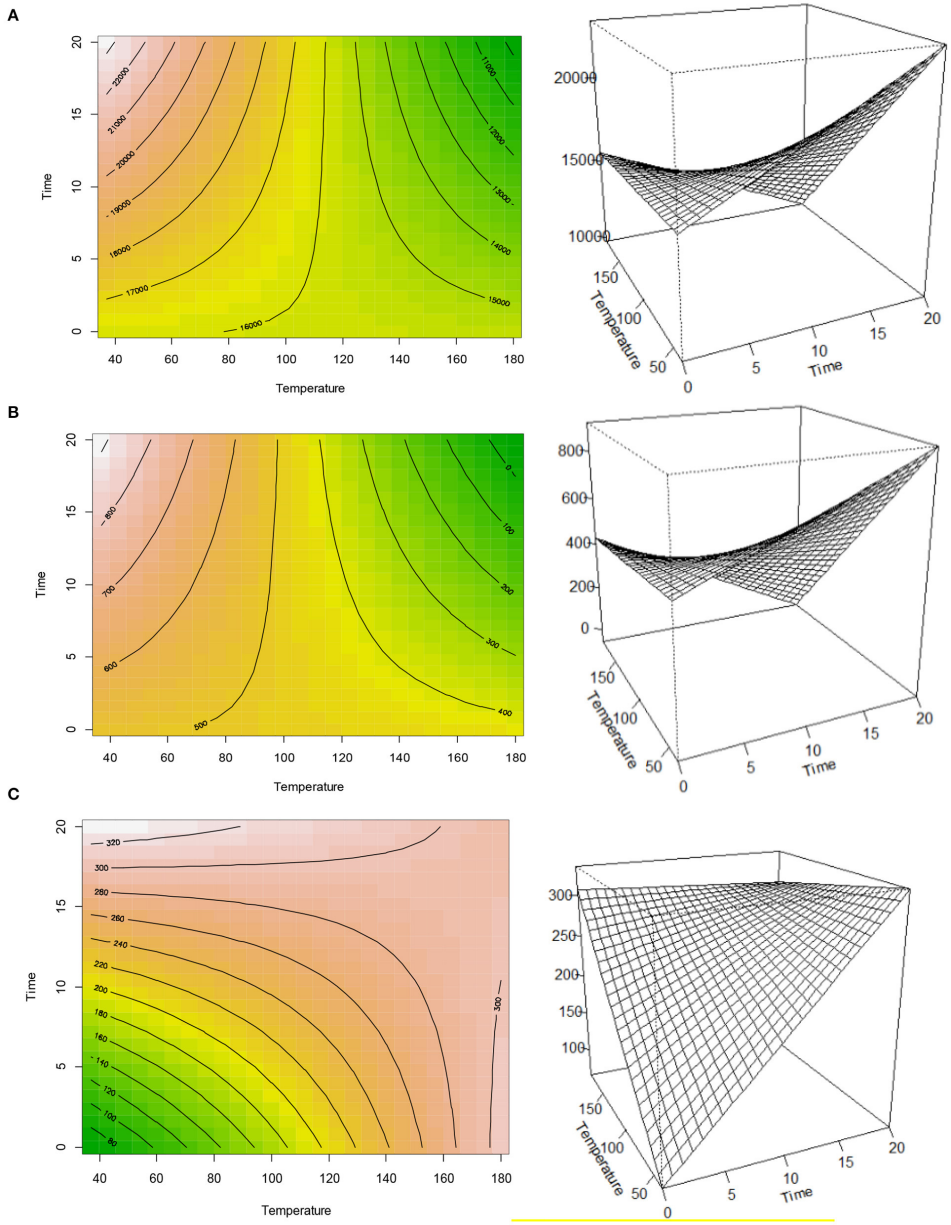
Contour-plots and response surface analysis of time (minutes)-temperature (°C) pre-treatment on sinapine **(A)**, sinapic acid **(B)**, and canolol **(C)**.

To establish the optimum pre-treatment time and temperature combinations for the major sinapates including sinapine, sinapic acid, and canolol (Figures 1A–C), contour-plots were plotted using response surface methodology. The contour-plots confirmed that both sinapine and sinapic acid concentrations decreased with increase in temperature and time (Figures 2A,B). However, an inverse trend was observed for canolol which increased with temperature (Figure 2C). The results further confirmed that sinapine and sinapic acid are the precursors of canolol at higher pressure and temperatures. These results were in agreement with previous results reported by Li and Guo (6) and Nandasiri et al. (9). Furthermore, with increase in pre-treatment time at higher temperatures, a reduction in the canolol concentration was observed. This reduction in canolol concentration over the time could be explained by the formation of dimers, oligomers and other degradation products when exposed for longer times at higher temperature pre-treatments (8).

Our previous studies showed that the optimum extraction condition for canolol using accelerated solvent extraction (ASE) was 160°C (9). However, the response surface analysis of contour-plots indicated the optimum extraction condition for canolol was between 160 and 180°C (Figure 2C). This was further confirmed by HPLC analysis where the highest canolol concentration was recorded at both 180°C for 5-min (427.11 ± 7.12 μg/g DW) and 160°C for 10-min (453.40 ± 17.66 μg/g DW) pre-treatment time (Table 1). Response surface analysis also indicated that the stationary point of response surface for canolol was located at 173.7°C at 17.12 min. These results showed it was possible to obtain a maximum amount of canolol using the above-described pre-treatment time-temperature combination. Further improvements in the modeling could assist industry to optimize the yield of canolol using the above time-temperature combination for dry heat extraction. Unfortunately, the extractability of both sinapine and sinapic acid, however, were outside the optimized conditions for the current analysis. Response surface analysis indicated that the stationary points for both the sinapine and sinapic acid were located at 115.81 and 101.15°C, respectively. Further modeling is required to obtain the optimized extraction conditions for both sinapine and sinapic acid.

**Table 1:** Effect of pre-treatment time and temperature on changes in major sinapates.

**Temperature (^**°**^C)**	**Time (min)**	**Sinapine (μg/g ^**ψ**^DW)**	**Sinapic acid (μg/g DW)**	**Canolol (μg/g DW)**
37°C		6667.52 ± 149.47	425.16 ± 7.58	135.63 ± 17.30
12°C	2	7641.86 ± 35.75	512.97 ± 20.67	164.43 ± 11.59
	5	7213.59 ± 102.47	504.53 ± 30.02	149.23 ± 14.24
	10	6725.75 ± 125.27	462.42 ± 7.27	229.97 ± 13.07
	15	6640.56 ± 113.27	434.23 ± 24.79	217.48 ± 10.57
	20	5985.38 ± 60.66	271.99 ± 15.12	239.67 ± 17.38
140°C	2	6837.13 ± 52.27	476.01 ± 9.42	153.06 ± 11.78
	5	6109.96 ± 207.10	440.29 ± 4.86	182.86 ± 2.65
	10	6445.03 ± 154.32	378.59 ± 19.79	320.65 ± 14.85
	15	5782.76 ± 103.93	303.89 ± 12.82	394.59 ± 23.72
	20	5296.80 ± 130.91	157.37 ± 12.90	404.82 ± 8.97
160°C	2	6427.37 ± 78.65	475.18 ±16.53	180.77 ± 9.82
	5	6499.69 ± 150.71	317.37 ± 4.99	351.97 ± 17.64
	10	5350.97 ± 60.46	259.45 ± 8.93	453.40 ± 17.66
	15	4667.24 ± 39.53	76.78 ± 7.34	353.01 ± 16.58
	20	4449.88 ± 147.75	74.41 ± 2.80	294.08 ± 17.73
180°C	2	7622.22 ± 181.27	360.19 ± 12.04	326.80 ± 9.70
	5	5870.31 ± 161.53	143.68 ± 17.90	427.11 ± 7.12
	10	4667.09 ± 79.23	75.62 ± 3.69	280.41 ± 15.38
	15	4577.56 ± 101.27	71.97 ± 6.23	221.85 ± 8.91
	20	4078.58 ± 81.42	65.96 ± 2.06	180.75 ± 6.24

*Results are expressed as mean values ± standard deviations*.

*Min, minutes; *μ*g, microgram; g, gram;*

ψ *DW, dry weight; °C, centigrade*.

In contrast, both sinapine and sinapic acid showed relatively higher correlation coefficient values with the response surface analysis. The adjusted *R*^2^-value for sinapine and sinapic acid was 0.88 and 0.77, respectively. Both the pre-treatment time and the interaction effect of time^*^temperature had a higher level of significance (*p* > 0.001) over the extractability of sinapine (Table 2). However, no significant impact was found in the pre-treatment temperature (*p* = 0.399). Likewise, for sinapic acid both the pre-treatment time (*p* > 0.05) and the interaction effect of time^*^temperature (*p* > 0.01) were significant. Similarly, pre-treatment temperature had a minimal impact (*p* = 0.338) on the extractability of the sinapic acid (Table 2). In contrast, canolol exhibited relatively lower correlation coefficient value with the response surface analysis. The adjusted *R*^2^-value of 0.16, indicated only temperature had an impact (*P* > 0.05) on the canolol concentration. Both pre-treatment time (*P* = 0.222), and time^*^temperature interaction (*P* = 0.295) showed no significance on the extractability of canolol (Table 2). This further suggests that extractability of canolol depended exclusively on the pre-treatment temperature.

**Table 2:** Response surface analysis of time-temperature pre-treatment on sinapine.

**RSM analysis**	**Estimate**	**STD error**	***t*-value**	**Significance**
**SP**				
Temp	−4.71	5.46	−0.86	0.399
Time	520.27	94.91	5.48	0.000
Time ^*^ Temp	−4.49	0.64	−7.00	0.000
*R*^2^-0.896				
Adj *R*^2^-0.877				
**SA**				
Temp	−0.67	0.68	−0.99	0.338
Time	31.18	11.85	2.63	0.017
Time ^*^ Temp	−0.31	0.08	−3.85	0.001
*R*^2^-0.801				
Adj *R*^2^-0.766				
**CL**				
Temp	1.70	0.78	2.17	0.044
Time	17.26	13.62	1.27	0.222
Time ^*^ Temp	−0.10	0.09	−1.08	0.295
*R*^2^-0.289				
Adj *R*^2^-0.164				

*DF, degrees of freedom; STD, standard; SP, sinapine; SA, sinapic acid; CL, canolol; Temp, Temperature; RSM, response surface analysis; R^2^, coefficient of correlation; Adj R^2^, adjusted coefficient of correlation*.

### Impact of Pre-treatments on Extractability of Phenolic Compounds

**Figure 3:**
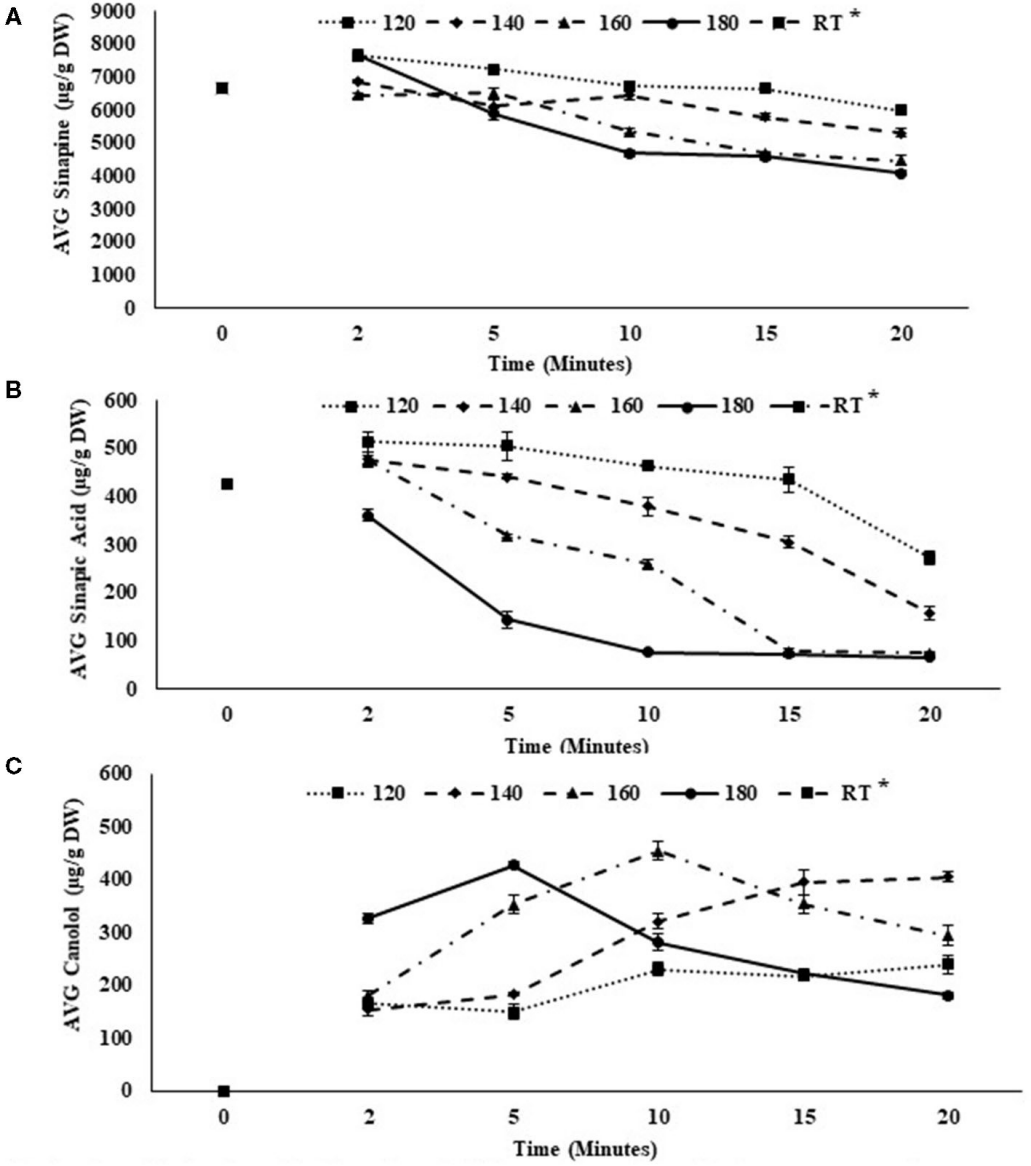
Changes in sinapine **(A)** sinapic acid **(B)** and canolol **(C)** concentration with the temperature-time pre-treatment for canola meal (* RT, Room Temperature).

The major sinapates present in canola meal, sinapine, sinapic acid and canolol are all thermolabile so that changes in concentrations depends on the pre-treatment and processing conditions (9, 22, 23). Consequently, the impact on individual compounds differs depending on the pre-treatment time and temperature (Figures 3A–C). In general, there was a decrease in the concentration of sinapine with increase in the treatment time (Figure 3A). The lowest sinapine concentration was observed at pre-treatment temperature of 180°C and a durations time of 20-min (4078.58 ± 81.42 μg/g DW) (Figure 3A). Pre-treatment at 120°C (7641.86 ± 35.75 μg/g DW) and 180°C (7622.22 ± 181.27 μg/g DW) showed the highest sinapine content with a treatment time of 2-min. Most interestingly the application of a pre-treatment temperature of 180°C reduced the sinapine concentration by 53% when the pre-treatment time was increased from 2- to 20-min (Table 1). Sinapine, is known to be the major flavor-active bitter tasting phenolic compound present in canola meal (24). Thus, the current finding would benefit the canola industry in two ways: by producing a low-bitter flavored canola by-product with a high economic value and concurrently producing a natural side stream phenolic rich extract with potential for the food/feed industry.

Similarly, sinapic acid concentration decreased with the increase in the pre-treatment temperature (Figure 3B). Thus, the optimum pre-treatment temperature for extraction of sinapic acid was around 120°C. Nevertheless, with the increase in the temperature as well as pre-treatment time there was a decrease in the sinapic acid concentration. Both 120°C for 2 min (512.97 ± 20.67 μg/g DW) and 120°C for 5 min (504.53 ± 30.02 μg/g DW) reported the highest sinapic acid concentration (Table 1). In sharp contrast, both 180 and 160°C pre-treatment temperatures at 10- (76.78 ± 7.34 μg/g DW) and 15-min (75.62 ± 3.69 μg/g DW) pre-treatment times showed the lowest sinapic acid concentration with no significant difference (*p* > 0.05; Table 1). This further suggests the extraction of canolol can be optimized between the pre-treatment temperatures of 160 and 180°C.

The results from the current study were consistent with previous findings that individual phenolic compounds were temperature dependent (6, 9), with the highest concentration of canolol recorded at 180°C (*P* > 0.05) for 5-min (427.11 ± 7.12 μg/g DW) and 160°C for 10-min (453.40 ± 17.66 μg/g DW) (Table 1). The inert pressurized atmosphere (N_2_) provided by RapidOxy® 100 during each pre-treatment produced higher yields of canolol compared to conventional extraction systems. The concentration of canolol, however, was found to decrease over a longer period of time at the higher temperatures. In sharp contrast, the lowest concentrations of canolol, was observed after a 2-min time period at 120, 140, and 160 (Figure 3C). These results suggest that longer exposure to higher temperatures, canolol is converted into other phenolic compounds (8). The degradation of canolol at temperatures above 180°C and elongated pre-treatment times (20 min) is attributed to its instability and conversion to dimers and oligomers as well as breakdown products (25). Apart from these hydroxycinnamic acid derivatives, other phenolic compounds including kaempferol, and kaempferol derivatives, and thermo generative phenolic compounds including thomasidioic acid (TA) are also impacted under the pressurized temperature processing conditions (4–7, 9).

### Structure-Based Phenolic Activity

**Figure 4:**
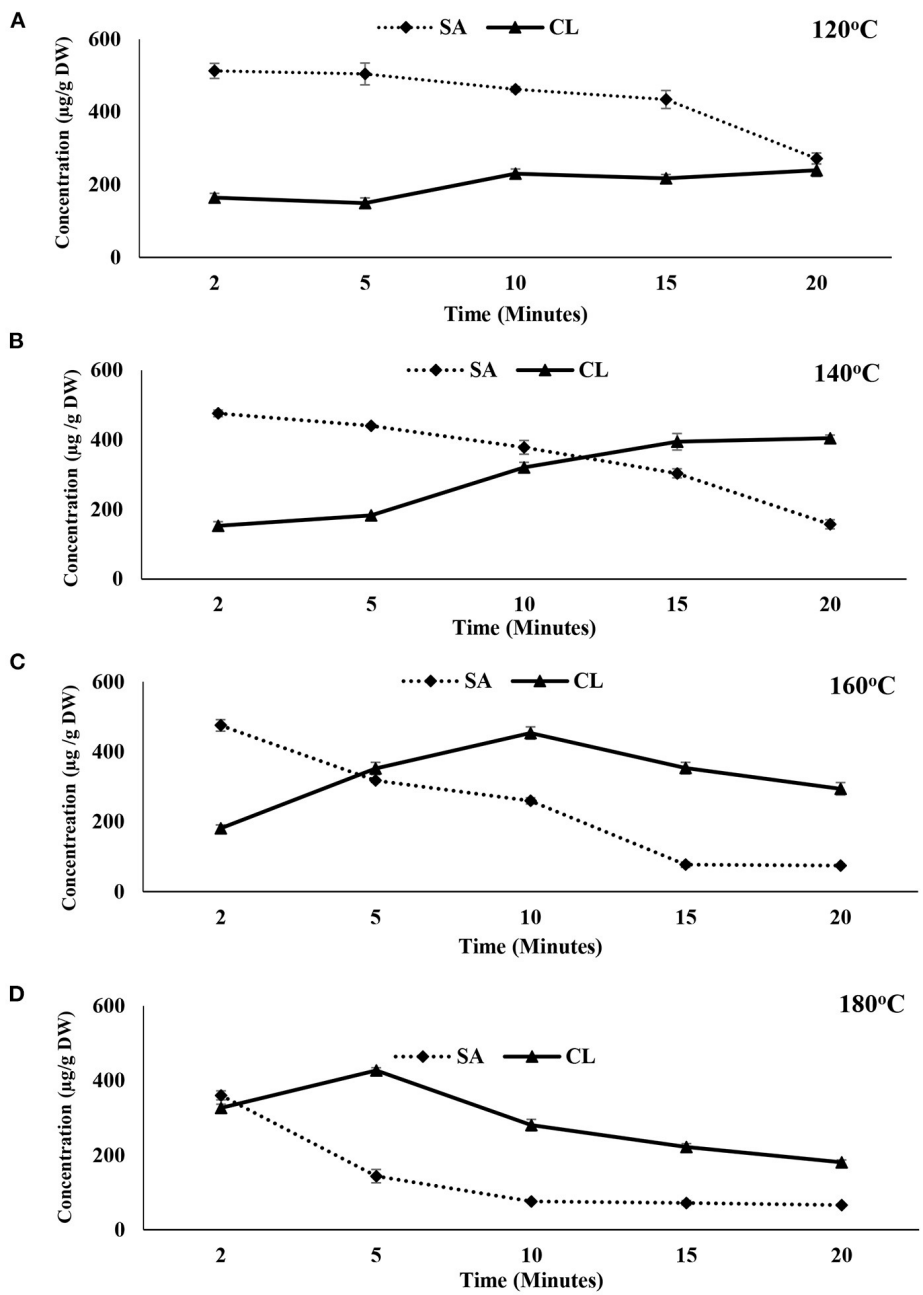
Structure-function relationship of sinapic acid and canolol for canola meal at **(A)** 120, **(B)** 140, **(C)** 160, and **(D)** 180°C.

Phenolic compounds have diverse structures often associated with different functions including color, flavor, redox potential, anti-mutagenic activity (25). High temperature processing is often associated with altering the chemical structures of phenolic compounds through breakage of different type of bonds while impacting the cellular matrix (26). Hence, it is essential to examine the structure-based activity between sinapic acid and canolol to better understand the effect of the pre-treatments. In fact, the results showed an inverse relationship between sinapic acid and canolol (Figures 4A–D) at different temperatures (120, 140, 160, and 180°C).

However, at the lower temperatures (120°C) sinapic acid concentration decreased gradually with the increase in the pre-treatment time while the corresponding concentration of canolol increased steadily (Figure 4A). An inverse relationship was observed for both sinapic acid and canolol at the processing temperature of 140°C (Figure 4B). At 20-min of pre-treatment time canolol showed the highest concentration while sinapic acid was at its lowest. Such changes in the concentration of canolol is directly associated with the degree of decarboxylation of sinapic acid at longer pre-treatment times (1). Nevertheless, with the increase in the treatment temperatures to 160 and 180°C, both compounds showed a decreasing pattern with the longer pre-treatment times (Figure 4C). The maximum canolol concentration was observed at both 160°C at 10 min (453.40 ± 17.66 μg/g DW) and 180°C at 5 min (427.11 ± 7.12 μg/g DW). At temperatures above 160°C, however, both sinapic acid and canolol decreased in their concentrations over time (Figures 4C,D).

Such changes could be attributed to structural alterations of canolol at higher temperatures. Both Harbaum-Piayda et al. (8) and Kraljić et al. (25) reported that canolol was converted into different forms including dimers and oligomers at their higher extraction temperatures. In addition, elevated temperatures over 140°C were reported to impact the concentration of the *cis*-isomer of sinapic acid (27). The *cis*-isomer was not detectable at temperatures of 160 and 180°C. Consequently, a decrease in both sinapic acid and canolol was observed at the higher temperatures.

**Figure 5:**
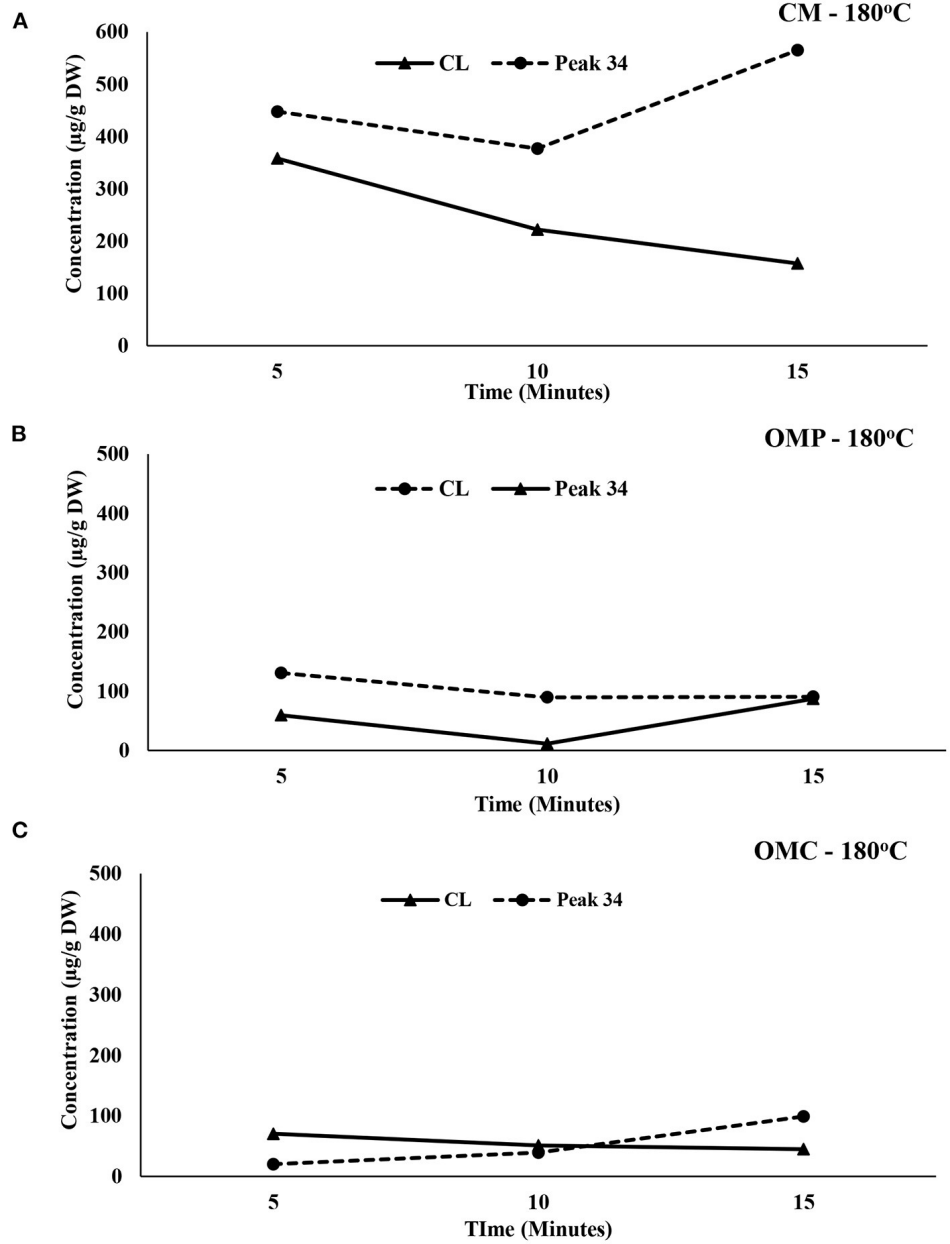
Structure-function relationship of canolol and peak RT-34 min for canola meal **(A)**, oriental mustard powder (OMP) **(B)**, and oriental mustard cake (OMC) **(C)** at 180°C.

To further understand the structure-based activity of canolol at higher temperatures an experiment was conducted at three different time points (5, 10, and 15 min) at 180°C pre-treatment time. HPLC analysis indicated that at higher temperatures with longer exposure times a novel phenolic compound was formed. Further, it was noted that there was an inverse relationship between canolol and the newly formed phenolic compound (Figure 5). Interestingly the novel phenolic compound (34.4 min) had an almost identical retention time to canolol (33.4 min). The structure-based activity of the novel phenolic compound was also examined in a different matrix using Oriental mustard powder and cake. All three samples canola meal (565.12 ± 11.07 μg/g DW), Oriental mustard cake (99.04 ± 8.11 μg SAE/g DW), and Oriental mustard powder (87.11 ± 14.00 μg SAE/g DW) showed the highest concentration of the novel phenolic compound after 15 min extraction time (Figures 5A–C). In contrast, the concentration of the novel compound was extremely low at the shorter pre-treatment times (5 min) suggesting that this compound could be a degraded product of canolol, or a novel phenolic compound formed at higher temperatures in the inert pressurized environment. However, further confirmation studies are needed.

### Qualitative Analysis of Phenolic Compounds

**Figure 6:**
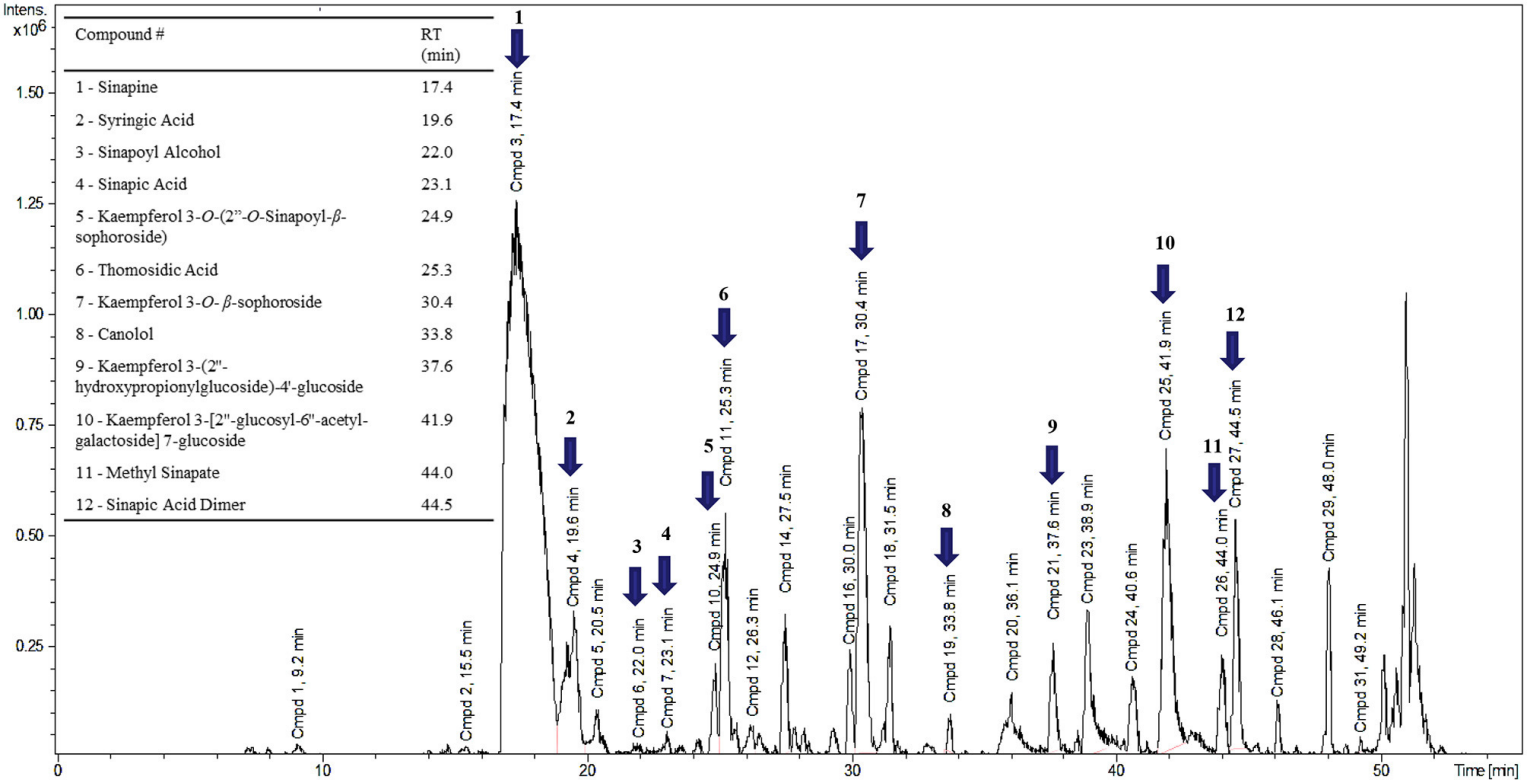
Liquid chromatogram of a representative canola meal extract pre heat treated at 160°C with tentative identification of phenolic compounds using LC-MS analysis (RT- retention time, min- minutes).

Only three major phenolic compounds were identified with the corresponding standards by HPLC. The lower sensitivity, lack of standards, similarity in UV-spectra and retention times between the different phenolic compounds limited the ability of HPLC to identifying other minor components present in the canola meal extracts (15, 28–30). Considering the above limitations of the HPLC, all interpreted signals were labeled as unknowns and subjected to mass spectrometry (MS) analysis for identification as described by Nandasiri et al. (13). Other key phenolic compounds were tentatively identified using the reference literature mass, fragmentation patterns and relative retention time (Figure 6). Apart from the major sinapates, nine other phenolic derivatives including syringic acid, methyl sinapates, thomasidioic acid, sinapic acid dimer, kaempferol 3-*O*-(2*α*-*O*-sinapoyl-*β*-sophoroside), and kaempferol 3-*O*-*β*-sophoroside were tentatively identified by liquid chromatography mass spectrometry (LC-MS/MS) (Figure 5). Further purification and fractionation studies are needed for the quantification of these phenolic compounds. Hence, further confirmation of the phenolic structures will require nuclear magnetic resonance (NMR) measurements.

## Conclusion

Using an inert environment, RapidOxy®100 proved an effective solvent-free dry-heat pre-treatment for enhancing the yield of phenolic compounds by ultrasonic extraction. Its compact and portable design and its ability to modify the gas supply holds considerable potential for enhancing bioactive compounds from underutilized agricultural by-products. To the best of the researchers' knowledge, this is the first report of the novel application of RapidOxy® 100 as a solvent-free pre-treatment prior to the extraction of phenolic compounds from canola and mustard.

## Data Availability Statement

The raw data supporting the conclusions of this article will be made available by the authors, without undue reservation.

## Author Contributions

RN came up with the concept and conducted all the experiments, results analysis, and manuscript writing. AI helped in the experiments with thermal analysis. UT-H and NE helped in writing and proofreading the manuscript.

## Conflict of Interest

The authors declare that the research was conducted in the absence of any commercial or financial relationships that could be construed as a potential conflict of interest.
